# *Tribolium castaneum* RR-1 Cuticular Protein TcCPR4 Is Required for Formation of Pore Canals in Rigid Cuticle

**DOI:** 10.1371/journal.pgen.1004963

**Published:** 2015-02-09

**Authors:** Mi Young Noh, Subbaratnam Muthukrishnan, Karl J. Kramer, Yasuyuki Arakane

**Affiliations:** 1 Department of Applied Biology, Chonnam National University, Gwangju, Korea; 2 Department of Biochemistry and Molecular Biophysics, Kansas State University, Manhattan, Kansas, United States of America; Howard Hughes Medical Institute, United States of America

## Abstract

Insect cuticle is composed mainly of structural proteins and the polysaccharide chitin. The CPR family is the largest family of cuticle proteins (CPs), which can be further divided into three subgroups based on the presence of one of the three presumptive chitin-binding sequence motifs denoted as Rebers-Riddiford (R&R) consensus sequence motifs RR-1, RR-2 and RR-3. The TcCPR27 protein containing the RR-2 motif is one of the most abundant CPs present both in the horizontal laminae and in vertical pore canals in the procuticle of rigid cuticle found in the elytron of the red flour beetle, *Tribolium castaneum*. Depletion of TcCPR27 by RNA interference (RNAi) causes both unorganized laminae and pore canals, resulting in malformation and weakening of the elytron. In this study, we investigated the function(s) of another CP, TcCPR4, which contains the RR-1 motif and is easily extractable from elytra after RNAi to deplete the level of TcCPR27. Transcript levels of the *TcCPR4* gene are dramatically increased in 3 d-old pupae when adult cuticle synthesis begins. Immunohistochemical studies revealed that TcCPR4 protein is present in the rigid cuticles of the dorsal elytron, ventral abdomen and leg but not in the flexible cuticles of the hindwing and dorsal abdomen of adult *T. castaneum*. Immunogold labeling and transmission electron microscopic analyses revealed that TcCPR4 is predominantly localized in pore canals and regions around the apical plasma membrane protrusions into the procuticle of rigid adult cuticles. RNAi for *TcCPR4* resulted in an abnormal shape of the pore canals with amorphous pore canal fibers (PCFs) in their lumen. These results support the hypothesis that TcCPR4 is required for achieving proper morphology of the vertical pore canals and PCFs that contribute to the assembly of a cuticle that is both lightweight and rigid.

## Author Summary

The insect cuticle is a remarkable biomaterial primarily formed from two different types of structural biopolymers, cuticular proteins and chitin. Despite a rather limited composition, insects produce diverse cuticles with the proper combination of mechanical properties such as strength, hardness and flexibility. Adult beetles are covered mostly by a hard cuticle, but they can fly because their cuticle is lightweight. The rigid cuticle is comprised of three major functional layers, namely the outermost envelope, the protein-rich epicuticle and the innermost chitin-protein rich procuticle. In addition, there are a large number of vertically oriented columnar structures denoted as pore canals that contain chitinous fibers (pore canal fibers) that are absent in soft and flexible cuticles. We have identified a cuticular structural protein, TcCPR4, which is predominantly localized in the pore canals of rigid cuticles of the red flour beetle. Loss of function of *TcCPR4* by RNA interference causes abnormal and amorphous pore canal fibers resulting in less organized pore canals that do not traverse the procuticle vertically. TcCPR4 plays a major role in determining the morphology of the vertical pore canals and pore canal fibers that contribute to the formation of a lightweight and rigid beetle cuticle.

## Introduction

Insects have a protective exoskeleton made up of a multi-layered cuticle that helps to withstand various environmental and pathogenic challenges. It consists of three morphologically and functionally distinct layers, the outermost waterproof envelope, the protein-rich epicuticle and the innermost chitin/protein-rich procuticle [1–3]. Structural cuticular proteins (CPs) and the matrix polysaccharide chitin are the primary components of the procuticle that consists of exo- and endocuticular layers. During cuticle maturation and tanning (sclerotization and pigmentation), some of the CPs are cross-linked by quinones or quinone methides produced by laccase 2-mediated oxidation of N-acylcatechols [4–6]. This vital process together with dehydration is important for insect development and growth because it produces the appropriate combination of mechanical and physical properties for different cuticles depending on the species and on specific regions of the insect anatomy even in the same animals [7,8]. The functions of individual insect CPs in determining the properties of the cuticle are not well understood. This study examines the role of a CP in *Tribolium castaneum* (red flour beetle), TcCPR4, which is present in rigid adult cuticles.

Recent progress in completion and annotation of the genome sequences of several insect species has revealed that there is a large number of genes encoding CP-like proteins in insect genomes [9–14]. Insect CPs are classified into several distinct families defined by the presence of specific amino acid sequence motifs [15,16]. The largest family is the CPR family, which includes proteins that contain a conserved amino acid sequence motif known as the Rebers & Riddiford (R&R) consensus sequence [17].

When amino acid sequences of proteins belonging to the CPR family are aligned, they fall into three groups, RR-1, RR-2 and RR-3, based on sequence similarity [18,19], and these groups tend to correlate with the type of cuticle (soft or hard) from which the proteins are derived. CPR proteins with the RR-1 motif have been found primarily in relatively soft and flexible cuticles, whereas many CPR proteins identified in rigid cuticles have a region of similarity called the extended RR-2 motif [20].

We recently reported the ultrastructure and morphology of rigid cuticle associated with the dorsal elytron, thoracic body wall and leg from pharate adults of *T*. *castaneum* [21]. Much like the larval body wall cuticle that is relatively soft and flexible in this species, elytral cuticle contains the envelope, epicuticle and procuticle that consists of numerous horizontally oriented chitin-protein laminae. However, unlike the larval body wall cuticle, there is a large number of vertical columnar structures in dorsal elytral cuticle, which extend directly from the apical plasma membrane protrusions of the underlying epidermal cells and penetrate the horizontal laminae, finally reaching the epicuticle. These vertical “pore canals” (PC) contain electron lucent fiber-like material denoted as “pore canal fibers” (PCF). Other regions of the body with rigid cuticle such as thoracic body wall and leg also have an ultrastructure very similar to that of the elytron. In contrast, there are fewer horizontal laminae and no vertical PCs with PCFs in the anatomical regions with soft, flexible and less pigmented cuticles such as the dorsal abdomen, ventral elytron and hindwing in mature adults of *T*. *castaneum* [21]. Apparently, this unique architecture and arrangement of numerous compact laminae and PCFs contribute to the formation of rigid cuticle.

TcCPR27 and TcCPR18 are two abundant cuticular proteins found in protein extracts of elytra from *T*. *castaneum* [8]. Both of these proteins belong to the RR-2 group of the CPR family. They are abundant in rigid cuticle such as the dorsal elytron, pronotum and ventral abdomen but are absent or constitute very minor components of soft and flexible cuticle such as those found in the dorsal abdomen and hindwing of *T*. *castaneum*. Furthermore, TEM immunogold labeling indicated that TcCPR27 protein is localized in both horizontal laminae and vertical pore canals in the procuticle of rigid cuticle [21]. Double-stranded RNA (dsRNA)-mediated gene silencing (RNAi) for *TcCPR27* resulted in an unorganized laminar architecture of the procuticle as well as abnormal and amorphous PCFs, which led to short, wrinkled and weakened elytra with the adults dying prematurely from dehydration approximately one week after eclosion [8,21].

In this study we identified another *Tribolium* protein, TcCPR4, which is more easily extractable from the elytra of TcCPR27-deficient adults. Unlike TcCPR18 and TcCPR27, TcCPR4 belongs to the RR-1 group of the CPR family and is also present in rigid cuticles of the elytron, ventral abdomen and leg but not in the flexible cuticles of the dorsal abdomen and hindwing. We analyzed the expression profile of the *TcCPR4* gene and localization of its protein product in cuticle by TEM immunogold labeling. RNAi was utilized to investigate the role(s) of *TcCPR4* in formation and stabilization of the rigid cuticles of *T*. *castaneum* adults.

## Results and Discussion

### Identification of the RR-1 protein, TcCPR4, in extracts of elytra from *T*. *castaneum*

**Fig 1:**
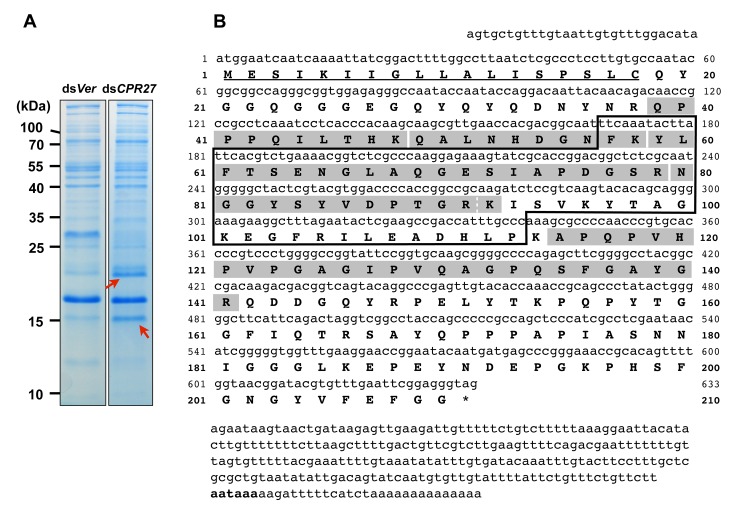
Identification of TcCPR4 protein in elytral cuticle of TcCPR27-deficient adult *T*. *castaneum*. (A) dsRNAs for *TcCPR27* (ds*CPR27*) and *TcVer* (ds*Ver*) (100 ng per insect) were injected into a mixture of penultimate instar and last instar larvae (n = 20). Proteins from the elytra of each dsRNA-treated newly emerged adults were extracted, and then the extracts were analyzed by SDS-PAGE. Two proteins with apparent molecular masses of 16 and 22 kDa (red arrows) were more extractable in TcCPR27-deficient elytra. (B) Nucleotide and deduced amino acid sequences of TcCPR4. The two proteins were cut out from the gel, digested with trypsin, and the resulting peptides were analyzed by MALDI-TOF mass spectrometry. Results were compared with conceptual trypsinization products of the computed proteome of *T*. *castaneum*. Both proteins exhibited the same six matched peptides that are highlighted in gray. The broken line between R^91^ and K^92^ indicates that there are two matched peptides, one is N^80^-R^91^ and the other is N^80^-K^92^. Predicted signal peptide is underlined. The box indicates the RR-1 motif. The potential polyadenylation signal (AATAAA) is indicated in bold.

We had previously identified two highly abundant cuticular proteins, TcCPR27 and TcCPR18, in elytral, thoracic and ventral abdominal cuticles that become highly sclerotized and pigmented in mature *T*. *castaneum* adults [8]. These two proteins are members of the CPR family that contain the RR-2 motif [15,17]. SDS-PAGE analysis of extracts of elytra from TcCPR27-deficient pharate adults (5 d-old pupae; ds*TcCPR27* RNA administered during late larval stages) revealed two additional major proteins with apparent sizes of 16 and 22 kDa based on their electrophoretic mobilities (red arrows in Fig. 1A). These two proteins were more abundant in the phosphate buffered saline (PBS)-extractable fraction than in similar PBS extracts prepared from ds*TcVer*-treated control (dsRNA for *T*. *castaneum Vermilion* (ds*TcVer*), a gene required for normal eye pigmentation [22], was injected as a negative control). To identify these proteins, each band was cut out from the gel, digested with trypsin and the resulting peptides were analyzed by MALDI-TOF mass spectrometry [8]. Comparison of those peptides with peptides derived from the conceptual trypsinization of the computed proteome of *T*. *castaneum* revealed one candidate gene responsible for both proteins (NP_001139387), denoted as *TcCPR4* (the numbering of CPs in *T*. *castaneum* does not reflect orthology with CPs in other species assigned the same number), which is also a member of the CPR family (CutProtFam-Pred: http://bioinformatics.biol.uoa.gr/CutProtFam-Pred/). Both proteins exhibited the same six matched peptides that are highlighted in gray in Fig. 1B. Peptide coverage for the mature TcCPR4 protein excluding the predicted signal peptide was 42%.

The cDNA sequence of the *TcCPR4* clone is identical that of the NCBI RefSeq gene prediction (Fig. 1B). The *TcCPR4* gene encodes a protein with 210 amino acid residues containing a putative signal peptide sequence (Fig. 1B) with a theoretical molecular weight and pI for the mature protein of 20.8 kDa and pH 6.2, respectively. The mature TcCPR4 protein contains the RR-1 cuticular protein motif rich in glycine (16.1%) and proline (11.5%), and there are no cysteines. It is, however, slightly atypical for an RR-1 protein scoring 16.4 when 35.0 appears to be the recommended score for an RR-1 protein based on profile hidden Markov models (CutProtFam-Pred) [9,16]. There is no α-helix, whereas six β-sheet sequences are predicted (PSIPRED: http://bioinf.cs.ucl.ac.uk/psipred/), four of which are in the RR-1 motif (F^57^-T^62^, A^68^-I^73^, S^78^-V^86^ and K^92^-T^98^). No N-glycosylation sites were predicted, whereas six O-glycosylation sites (S^135^, T^153^, T^159^, T^165^, S^167^ and S^178^) were (NetNGlc 1.0 and NetOGlc 4.0: http://www.cbs.dtu.dk/services/). The *TcCPR4* gene maps to linkage group 1 = X of the *T*. *castaneum* genome (BeetleBase: http://beetlebase.org).

### Developmental pattern of expression of *TcCPR4*

Real-time PCR was performed to analyze the expression pattern of *TcCPR4* during development. Few or no transcripts for *TcCPR4* were detected at the embryonic, larval, pharate pupal or adult stages of development (S1A Fig.). However, the transcript level of the *TcCPR4* gene dramatically increased in 3 d-old pupae and declined soon thereafter by the time of adult eclosion (5 d-old pupae) (S1B Fig.).

We previously identified two other highly abundant cuticular proteins, TcCPR27 and TcCPR18, in extracts of rigid cuticle of adult *T*. *castaneum* [8]. Unlike *TcCPR27* and *TcCPR18* whose transcripts were highest in 4 d-old pupae [8], *TcCPR4* mRNA expression was highest one day prior to the peak abundance of the former two genes.

### Immunohistochemistry

To localize the TcCPR4 protein in adult cuticle, immunohistochemistry was performed with the TcCPR4 antibody. First, the specificity of the antibody was confirmed by western blot analysis of protein extracts of elytra dissected from ds*TcVer*- and ds*TcCPR4*-treated insects collected on pupal day 5. The TcCPR4 antibody detected two proteins (arrows in S2 Fig.) with apparent molecular weights corresponding to the same two proteins that were more extractable in TcCPR27-deficient insects (red arrows in Fig. 1A) than in ds*TcVer* and ds*TcCPR4* extracts. This result suggested that the larger protein (~22 kDa) corresponds to the mature TcCPR4 protein and the smaller protein (~16 kDa) is a truncated form of TcCPR4.

**Fig 2:**
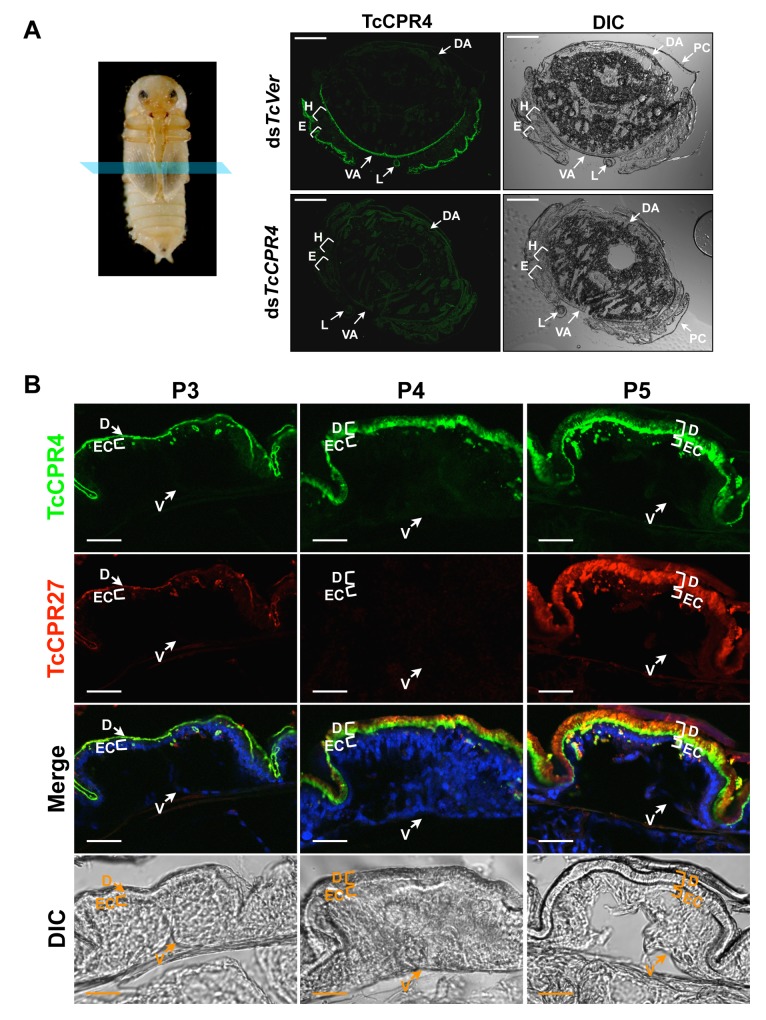
Localization of TcCPR4 protein in adult cuticles of *T*. *castaneum*. (A) Immunohistochemical analysis was performed to determine the locations of TcCPR4 in adult cuticles. Cryosections of pharate adults (5 d-old pupae) were incubated with the anti-TcCPR4 antibody, which was then detected by Alexa Fluor 488 goat anti-rabbit IgG (green). E: elytron, H: hindwing, VA: ventral abdomen, DA: dorsal abdomen, L: leg, PC: pupal cuticle. Scale bar = 200 μm. (B) Immunolocalization of TcCPR4 and TcCPR27 in elytral cuticle. Cryosections of 3 d-, 4 d- and 5 d-old pupae were incubated with the anti-TcCPR4 or anti-TcCPR27 antibody [21]. Anti-TcCPR4 and anti-TcCPR27 antibodies were detected by Alexa Fluor 488 goat anti-rabbit IgG (green) and Alexa Fluor 546 rabbit anti-chicken IgG (red), respectively. Nuclei were stained with To-Pro-3 (blue). D: elytral dorsal cuticle, V: ventral elytral cuticle, EC: epithelial cell. Scale bar = 20 μm.

In pharate adults (5 d-old pupae), TcCPR4 protein was detected in cuticles of the dorsal side of the elytron, thoracic body wall, ventral abdomen as well as leg, all of which become more rigid and darker as the adult matures (Fig. 2). Little or no TcCPR4 immunoreactivity was detected in soft, membranous cuticles such as the ventral side of the elytra, hindwing or dorsal abdomen (Fig. 2). These results are consistent with what has been observed in an enhancer trap line, KS217, in which a piggyBac element was inserted in a gene encoding TcCPR4 [23]. In KS217 pharate adults (5 d-old pupae), enhanced green fluorescent protein (EGFP) is expressed in the cuticles of elytron, head, pronotum, ventral abdomen and leg, all of which become highly sclerotized and pigmented in the mature adult but not in flexible and less pigmented cuticles of the dorsal abdomen and hindwing (S3 Fig.). It has been suggested that RR-1 and RR-2 proteins are associated with relatively flexible and rigid cuticles, respectively [20]. However, it has also been suggested that the former proteins are predominant in endocuticle (post-ecdysial) and that the later proteins occur mainly in exocuticle (pre-ecdysial), irrespective of the cuticle’s physical properties [19]. By contrast, our results demonstrate that an RR-1 protein, TcCPR4, is exclusively localized to a rigid cuticle and is in the pre-ecdysial exocuticle.

In the elytron, TcCPR4 protein was readily detected in the dorsal cuticle of 3 d-old pupae, while a high level of the most abundant rigid adult cuticle protein, TcCPR27, was detected in 4 and 5 d-old pupae (Fig. 2B), results consistent with the expression profiles of transcripts for these two genes (S1 Fig.) [8]. We recently reported that TcCPR27 protein was present throughout the procuticle in both the horizontal, compact chitinous laminae and the vertical columnar structures, but not in the epicuticle or envelope layers of dorsal elytral cuticle as demonstrated by immunogold labeling and transmission electron microscopy (TEM) [21]. Dual immunostaining for TcCPR4 and TcCPR27 proteins indicated that both proteins are present in the procuticle. However, the TcCPR4 protein was detected predominantly in the inner regions of procuticle of the elytron, whereas it was deficient in the upper region of the procuticle relative to TcCPR27, which was uniformly abundant in the entire procuticle (Fig. 2B; see panel marked “Merge”). Similar protein localizations of both TcCPR4 and TcCPR27 proteins were also observed in rigid thoracic body wall cuticle (S4 Fig.).

### TEM and immunogold labeling of TcCPR4 in rigid adult cuticle

**Fig 3:**
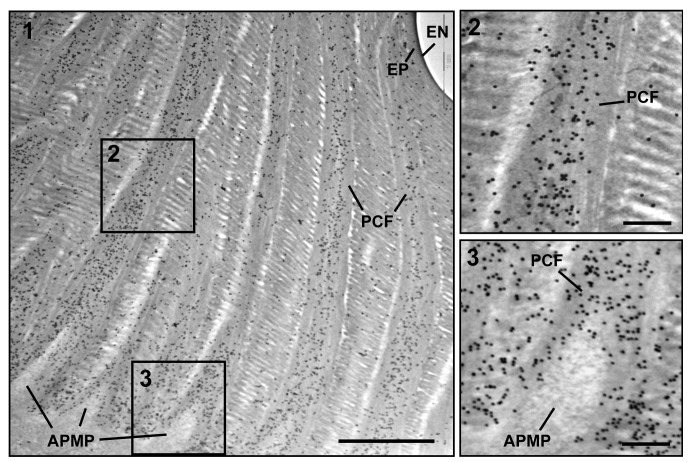
Immunogold labeling followed by TEM analysis for TcCPR4. Ultra-thin sections (~90 nm) of pharate adults (5 d-old pupae) were incubated with anti-TcCPR4 antibody. Anti-TcCPR4 antibody was detected using goat anti-rabbit IgG conjugated to 10 nm gold particles. The pore canal with pore canal fibers (PCF) in their core (2) and apical plasma membrane protrusions (APMP) (3) were enlarged. Scale bar in panel 1 = 1 μm and in panels 2 and 3 = 200 nm.

Insect cuticle is composed of several morphologically and functionally distinct layers such as the envelope, epicuticle and procuticle [1–3]. As we have reported previously, the envelope, epicuticle and procuticle consisting of numerous horizontally oriented chitinous laminae parallel to the epidermal cell apical plasma membrane are easily identifiable in dorsal elytral cuticle from the pharate adult (5-day old pupae) of *T*. *castaneum* (Fig. 3) [21]. There are also numerous vertical columnar structures denoted as pore canals (PCs) containing long vertically oriented fibers (PCFs) that extend directly from the apical plasma membrane protrusions (APMP in Fig. 3) of the underlying epidermal cells and penetrate the horizontal laminae vertically, reaching all the way to the epicuticle. Similar vertical fibrillar structures or vertical fibrils have been observed not only in insects [24–26] but also in the exoskeletons of crustaceans, such as *Homarus americanus* (American lobster), *Callinectes sapidus* (Atlantic blue crab) and *Tylos europaeus* (sand-burrowing isopod) [27–29] after removal of minerals and proteins. In *T*. *castaneum*, other regions with rigid cuticles such as thoracic body wall and leg exhibit an ultrastructure very similar to that of the elytron’s dorsal cuticle (see panels A, E, I and M in S8 Fig.), but there are fewer horizontal laminae and no vertical PCFs in the regions with soft, flexible and less pigmented cuticles such as the dorsal abdomen, ventral elytron and hindwing [21]. These observations suggested that the dense arrangement of numerous compact laminae and PCFs contribute to not only to the architecture but also to the mechanical strength of rigid cuticle.

To achieve a more precise localization of the TcCPR4 protein in rigid cuticle of *T*. *castaneum*, we performed immunogold labeling followed by TEM analysis. In elytra from pharate adults (5 d-old pupae), TcCPR4 protein was predominately localized together with the PCFs as well as in the vicinity of the APMPs, but it was much less abundant in the horizontal laminae and essentially absent in the epicuticle and envelope layers (Fig. 3). As observed previously with TcCPR27 [21], the association of TcCPR4 protein with the fiber-like structures surrounding the APMPs (Figs. 3 and S5) suggested that both of these proteins in the PCFs originate from the APMPs.

### dsRNA-mediated loss of function of TcCPR4

**Fig 4:**
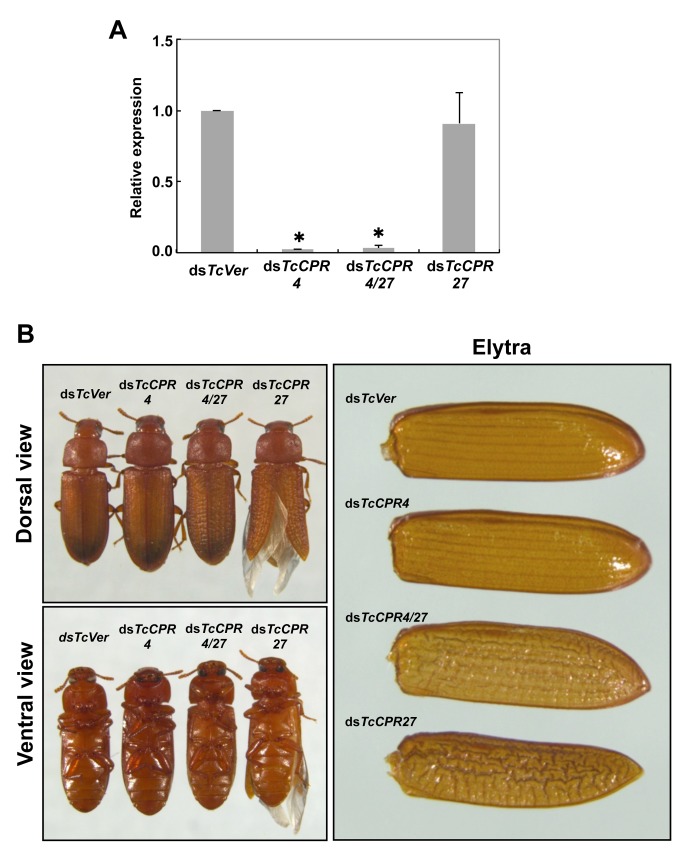
Injection of ds*TcCPR4* prevents severe elytral morphological defects produced by RNAi for *TcCPR27*. dsRNAs for *TcCPR4*, *TcCPR27*, *TcCPR4/27* and *TcVer* (100 ng per insect) were injected into late instar larvae (n = 40). (A) The expression of *TcCPR4* was analyzed by real-time PCR. cDNAs were prepared from total RNA isolated from 5 d-old pupae (n = 3). Expression levels of *TcCPR4* are presented relative to the levels in ds*Ver*-injected control insects. The transcript levels of *T*. *castaneum* ribosomal protein S6 (*TcRpS6*) were measured to normalize for differences in the concentrations of cDNA templates between samples. An asterisk indicates a significant difference in transcript levels of *TcCPR4* between control and test insects (p < 0.05, t-test). Data are shown as mean ± SE (n = 3). (B) Insects treated with those dsRNAs developed and grew normally. The ds*TcCPR4*-treated adults exhibited elytra indistinguishable from those of ds*TcVer*-treated control insects. Elytra from the resulting ds*TcCPR27*-treated adults were malformed, wrinkled, warped and fenestrated as reported previously [8], while those from the resulting ds*TcCPR4/27*-treated adults were less severely affected and expanded well enough to yield a decrease in desiccation-induced mortality produced by *TcCPR27* RNAi (see S7 Fig.).

RNAi was used to investigate the function(s) of *TcCPR4*. Injection of dsRNA for *TcCPR4* (ds*TcCPR4*) led to a substantial decrease in mRNA of the *TcCPR4* gene (Fig. 4A). In addition, the abundance of gold particles as well as the observation that TcCPR4 immunostaining is strongly reduced in dorsal elytral cuticle after injection of ds*TcCPR4* (S5B–S6 Figs.) demonstrates a depletion of TcCPR4 at the protein level and also a high specificity of the antibody used to detect the TcCPR4 protein. Similarly, administration of a mixture of dsRNAs for *TcCPR4* and *TcCPR27* (ds*TcCPR4/27*) significantly reduced the transcript levels for both genes to about the same extent as in animals treated with individual dsRNAs (Fig. 4A).

TcCPR4 protein is co-localized with chitin in rigid cuticles such as the dorsal elytron and thoracic body wall. Chitin staining in TcCPR4-deficient pharate adults, however, was indistinguishable from the level observed in ds*TcVer*-treated control animals, indicating that the lack of TcCPR4 has no effect on the cuticular chitin content (S6 Fig.). Administration of ds*TcCPR4* during late larval instars had no apparent effect on larval and pupal development with the resulting adults developing and growing normally. In addition, their elytra had a normal shape and length (Fig. 4B).

Loss of function of *TcCPR27* caused by RNAi resulted in a terminal adult phenotype with malformed and abnormal elytra as reported previously [8]. Lethality was apparently due to dehydration that resulted from failure of the misshapen elytra to cover the membranous dorsal abdomen and to seal it against trans-cuticular water loss. Indeed, manual excision of the distal half of the elytron from a mature wild-type adult also led to high mortality [8]. The elytra from TcCPR27-deficient adults were wrinkled, bumpy, warped and fenestrated, and did not fully cover their entire abdomen (Figs. 4B and S7). Interestingly, the elytra of TcCPR4/27-deficient adults exhibited less severe morphological defects than those of ds*TcCPR27*-treated adults (Figs. 4B and S7). Although the elytra of ds*TcCPR4/27* adults had a rough surface and some of them were split apart compared to those of ds*TcVer*-control animals, they elongated to cover the abdomen well enough to decrease the dehydration-induced high mortality produced by injection of ds*TcCPR27* (Figs. 4B and S7).

### Mislocalization of TcCPR4 protein in TcCPR27-deficient adult cuticle

**Fig 5:**
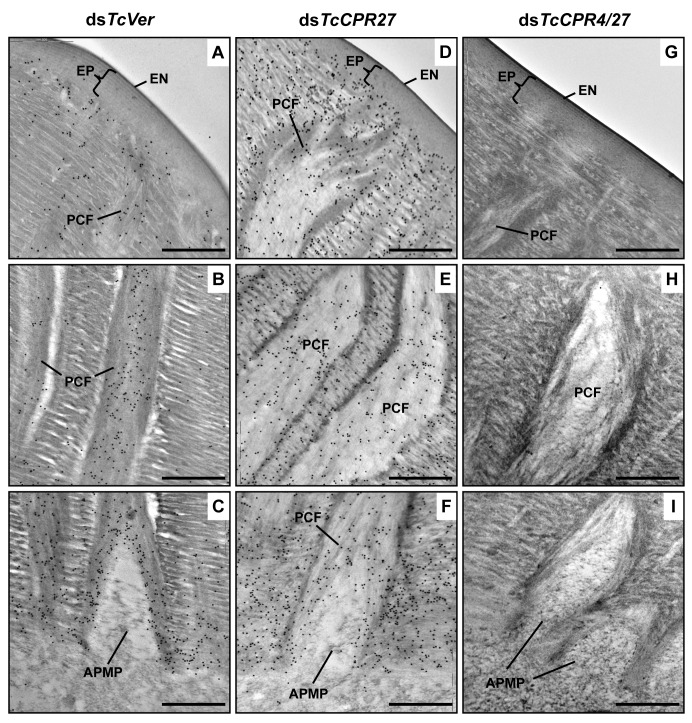
Localization of TcCPR4 protein in elytral cuticle from TcCPR27-deficient insects. Ultra-thin sections (~90 nm) of pharate adults (5 d-old pupae) that had been injected with dsRNA for *TcCPR27*, *TcCPR4/27* (co-injection) or *TcVer* (100 ng per insect) in late instar larvae were incubated with anti-TcCPR4 antibody. Anti-TcCPR4 antibody was detected by goat anti-rabbit IgG conjugated to 10 nm gold particles. TcCPR4 protein is mainly present in vertical PCFs of elytral cuticle from ds*TcVer*-treated insects (A-C), while it is distributed in the entire procuticle of the elytra from TcCPR27-deficient insects (D-F). The number of gold particles is drastically decreased in TcCPR4/27-deficient insects (G-I). EN: envelope, EP: epicuticle, PCF: pore canal fibers, APMP: apical plasma membrane protrusion. Scale bar = 500 nm.

Interestingly, depletion of TcCPR27 resulted in mislocalization of the TcCPR4 protein. TcCPR4 protein was mainly associated with PCFs and regions around the APMPs in elytra of ds*TcVer*-treated control insects (Figs. 3, 5A-5C and S5), whereas it was distributed over the entire procuticle including the horizontal laminae in TcCPR27-deficient elytra (Fig. 5D-5F). The results suggested that the presence of TcCPR27 protein in elytral cuticle is critical for the unique localization of TcCPR4 protein. A higher yield of TcCPR4 in the protein extracts of elytra from ds*TcCPR27*-treated pharate adults (Fig. 1) may be due to this mislocalization. As was observed in elytra of ds*TcCPR4*-treated insects, the abundance of gold particles was drastically decreased in elytra from TcCPR4/27-deficient insects (Figs. 5G-5I and S5B).

**Fig 6:**
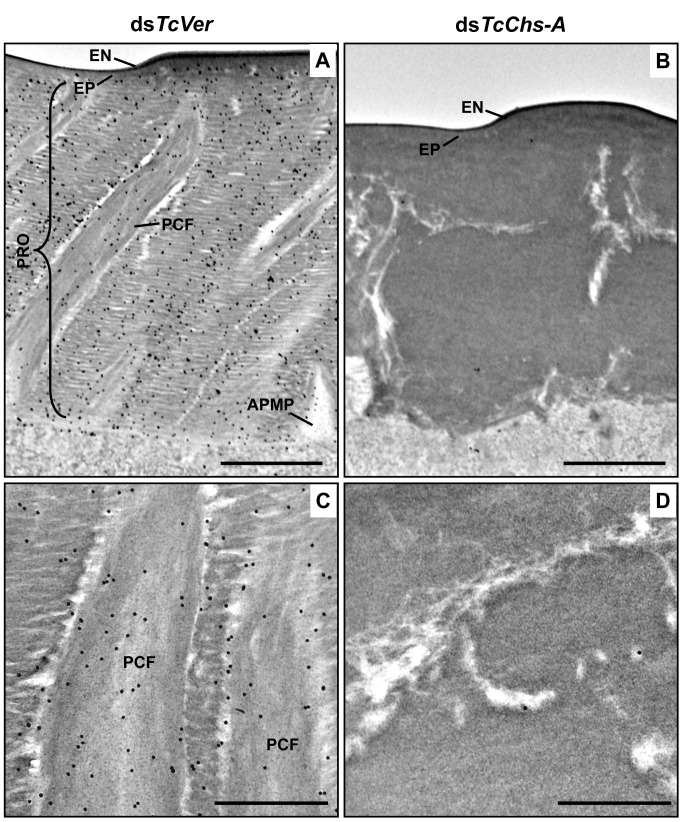
Wheat germ agglutinin (WGA)-gold labeling TEM of dorsal elytral cuticle of *T*. *castaneum*. Ultra-thin sections (~90 nm) of pharate adults (5 d-old pupae) that had been injected ds*TcVer* (200 ng per insect) in late instar larvae were incubated with gold-labeled (10 nm) WGA (EY Laboratories) to detect chitin in the dorsal side of elytral cuticle. dsRNA for *chitin synthase-A* (ds*TcChs-A*) which is required for cuticular chitin synthesis [30] was injected as a negative control. Chitin was detected in both horizontal laminae and vertical PCFs of elytral cuticle from ds*TcVer*-treated insects (A and C). Few or no gold particles were observed in TcChs-A-deficient insects (B and D). EN: envelope, EP: epicuticle, PRO: procuticle, PCF: pore canal fibers, APMP: apical plasma membrane protrusion. Scale bar in A and B = 1 μm and C and D = 500 nm.

Like the horizontal laminae, the vertically oriented PCFs appear to be made of chitin as they bind to gold-labeled wheat germ agglutinin. The gold particles were detected in both horizontal laminae and PCFs in the dorsal elytral procuticle from ds*TcVer*-treated control insects (Fig. 6A and 6C), whereas little or no particles were detected in insects that had been injected dsRNA for *TcChs-A*, the enzyme required for cuticular chitin synthesis [30] (Fig. 6B and 6D). The dorsal elytral cuticle from the latter insects exhibited a malformed and amorphous architecture lacking both horizontal laminae and vertical PCs.

The findings reported here indicate that TcCPR4 is associated with chitin fibers in the pore canals and that this process requires the presence of TcCPR27. We postulate that in the absence of TcCPR27, TcCPR4 diffuses throughout the procuticle including into the horizontal laminae made of chitin and protein and it is not confined only to the pore canals. We further postulate that the unique vertical arrangement of chitin fibers in the pore canals is due to an interaction (perhaps covalent cross-linking) between TcCPR27 and TcCPR4 when binding to chitin. Since TcCPR27 is also found outside of the PCs [21], the compact organization of chitin fibers within the pore canals is probably due to an interaction of the chitin fibers with TcCPR4 and perhaps TcCPR27.

### Ultrastructure of elytral cuticle from ds*TcCPR4*-treated and ds*TcCPR4/27*-treated insects

**Fig 7:**
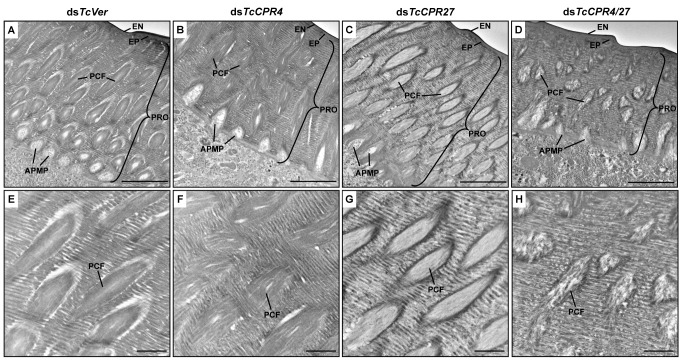
Ultrastructure of elytral cuticle of TcCPR4-, TcCPR27- and TcCPR4/27-deficient pharate adults. Elytra from pharate adults (5 d-old pupae) that had been injected with ds*TcCPR4*, ds*TcCPR27*, ds*TcCPR4/27* and ds*TcVer* into late instar larvae were collected for analysis of ultrastructure by TEM. Panels E-H show enlarged images of the horizontal laminae and vertical pore canals in the procuticle of the dorsal elytral cuticles from each set of dsRNA treated-insects. EN, envelope; EP, epicuticle; PRO, procuticle; PCF, pore canal fiber; APMP, apical plasma membrane protrusion. Scale bar in A-D = 2 μm and E-H = 500 nm.

The elytra from TcCPR4- and TcCPR4/27-deficient pharate adults (5 d-old pupae) were collected and used to analyze morphological and ultrastructural changes. The elytra from ds*TcVer*-treated control pharate adults exhibited horizontal electron-dense chitin-protein laminae and vertical pore canals containing a core of electron lucent PCFs that penetrate vertically through the entire procuticle (Fig. 7A and 7E). In addition, as reported previously [21], electron lucent spacing at the boundary between PCFs and the horizontal laminae was evident (Fig. 7A and 7E). Unlike the pore canals in the dorsal cuticle of the elytron following ds*TcVer* RNAi, those of ds*TcCPR4*-treated insects appeared to be less organized and irregular and did not traverse the procuticle in a straight line as the control pore canals (Fig. 7B and 7F). There was no electron lucent boundary between the PCs and the horizontal laminae in the elytron from ds*TcCPR4*-treated insects presumably because the abnormal and amorphous PCFs were less compact and swollen, filling the entire pore canal lumen (Fig. 7B and 7F). Similar ultrastructural defects were observed in other body regions with rigid cuticles such as thoracic body wall and leg (S8 Fig.). These results suggested that TcCPR4 plays a critical role in determining the morphology and ultrastructure of PCFs and pore canals in rigid adult cuticle of *T*. *castaneum*.

We previously reported that depletion of TcCPR27 protein by RNAi resulted in an unorganized laminar architecture and amorphous PCFs, which produced short, wrinkled and weakened elytra [21]. The horizontal laminae and vertical pore canals in the procuticle of elytra from ds*TcCPR27*-treated insects were more electron-lucent compared to those of ds*TcVer*-treated insects (Figs. 5D-5F, 7C and 7G). In contrast, the boundary separating the vertical PCFs embedded in the horizontal laminae in those cuticles from TcCPR27-deficent insects becomes more electron dense (Figs. 5D-5F, 7C and 7G). TcCPR18, the second most abundant cuticular protein in elytra [8], may contribute to this enhanced electron density because the boundary of the PCFs is not evident in elytra from ds*TcCPR27*/*18*-treated insects [21].

It was of interest to study the ultrastructure of elytral cuticle from TcCPR4/27-deficient insects since injection of ds*TcCPR4/27* resulted in a less severe elytral defect compared to that of the ds*TcCPR27* treatment (Figs. 4B and S7). RNAi of both *TcCPR4* and *TcCPR27* genes, however, exhibited the electron-lucent, unorganized horizontal laminae compared to those of the ds*TcVer*-treated control. In addition, the shape of the vertical pore canals was quite abnormal, exhibiting amorphous fibrous material in their lumen (Figs. 5G-5I, 7D and 7H). Although we have not elucidated how depletion of both TcCPR4 and TcCPR27 protein by RNAi leads to less severe elytral cuticle defects than that of ds*TcCPR27*-treated insects, it is possible that mislocalization of the TcCPR4 protein produced by RNAi for *TcCPR27* affects the expansion of the elytra, which occurs shortly after eclosion, resulting in shorter, wrinkled and warped elytra compared to those from the ds*TcVer*-treated control as well as ds*TcCPR4/27*-treated insects. An alternative hypothesis involves a geometrical consideration of the cuticle. Qiao et al. [31] reported that BmorCPR2 protein, which contains the RR-1 motif, is responsible for the phenotype of the *Bombyx mori stony* mutant larvae, which have a malformed body with reduced cuticle tensile properties. In general, insects need to have rigidity or flexibility of the cuticle in all three dimensions. If one dimension is compromised without affecting the others, then the cuticle may become more flexible or stiff, allowing it to cope better or worse with the cuticle expansion process. This hypothesis may help to explain why TcCPR4 is not found in soft cuticles where there are few if any pore canals with PCFs in their core. Elytra from TcCPR4/27-deficient adults were significantly thinner than control elytra (mean ± SE of ds*TcVer* and ds*TcCPR4/27* are 6.29 ± 0.58 and 3.70 ± 0.70 (μm), respectively. p-value = 5.1E-07) (S9 Fig.), softer and more fragile when handled with forceps in comparison to those of ds*TcVer* control insects. This flexibility may allow full expansion of the elytra after eclosion, resulting in a more normal appearance of their surface even though the ultrastructure and mechanical properties are aberrant.

**Fig 8:**
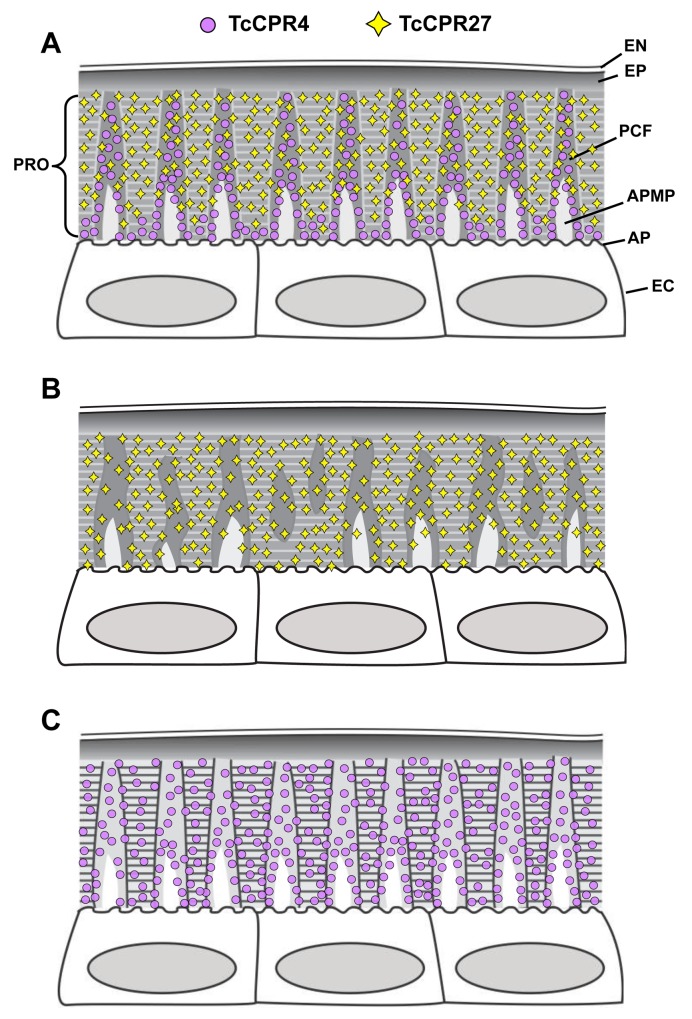
Schematic diagram of structure and localization of TcCPR4 and TcCPR27 proteins in rigid cuticle of adult *T*. *castaneum*. The body regions with highly sclerotized and pigmented cuticle such as the dorsal elytron, thoracic body wall and legs of *T*. *castaneum* adults are composed of the envelope (EN), epicuticle (EP) and procuticle (PRO). In the procuticle, there are numerous chitinous horizontal laminae and vertical pore canals with pore canal fibers (PCFs) in their core that directly extend from the apical plasma membrane protrusions (APMP) to the epicuticle. TcCPR27 protein is present in both the horizontal laminae and vertical PCFs, whereas TcCPR4 is predominantly localized in the latter structure. PC, pore canal; AP, apical plasma membrane; EC, epithelial cells underlying the rigid cuticle. A. Wild type, B. *TcCPR4* RNAi. C. *TcCPR27* RNAi.

In summary, we have identified in the red flour beetle a cuticle structural protein, TcCPR4, which belongs to the RR-1 group of the CPR family. It is found in the rigid dorsal cuticle of the elytron as well as in other tissues with rigid cuticles such as the ventral abdomen and leg but not in soft cuticles such as those in the hindwing and dorsal abdomen. In the rigid cuticles of adult *T*. *castaneum* including the dorsal elytral cuticle, there are not only numerous horizontal laminae but also a large number of vertical pore canals with PCFs in their core. This distinctive cuticle ultrastructure is only found in body regions that harden and darken in mature *T*. *castaneum* adults. The TcCPR4 protein is present predominantly in PCFs and the region around the APMPs that appear to be the sites where the vertically oriented pore canals originate. A schematic representation of the alterations in cuticle morphology after RNAi of either the *TcCPR4* or *TcCPR27* gene is shown in Fig. 8. Depletion of TcCPR4 protein by RNAi caused an abnormal shape of the pore canals, resulting in amorphous and less compact PCFs that fill the lumen. These results indicate that TcCPR4 has an important role in determining the morphology of the vertical pore canals and PCFs that contribute to the formation of a lightweight and rigid beetle cuticle.

## Materials and Methods

### Insects

The GA-1 strain of *T*. *castaneum* was reared in 50% relative humidity at 30°C under standard conditions [32].

### Protein extraction and identification

Elytra of TcCPR27-deficient 5 d-old pupae (n = 5) were dissected and homogenized in 120 μl of cold phosphate-buffered saline (0.01 M PBS, pH 7.4) containing a proteinase inhibitor cocktail (Thermo Scientific). The homogenate was centrifuged at 13,000 x g for 2 min at 4°C. The supernatant was collected as the PBS soluble fraction. The pellet was resuspended in 120 μl of SDS-PAGE sample buffer and then heated at 95°C for 10 min followed by centrifuged at 13,000 x g for 2 min. The supernatant was collected as SDS-PAGE soluble fraction. Those protein extracts were analyzed by 15% SDS-PAGE. After staining the gels with Commassie Blue G-250, the protein band selected was excised and digested with trypsin, and the resulting fragments were analyzed by MALDI-TOF mass spectrometry as described previously [8].

### Cloning a full-length *TcCPR4* cDNA

Primers that included the predicted start and stop codons were used to amplify the coding sequence for *TcCPR4* (633 bp) from first strand cDNA synthesized from total RNA extracted from pupae (mixture of 0 d- to 5 d-old pupae). The PCR product was cloned into pGEM-T vector (Promega) and sequenced. To obtain the near full-length *TcCPR4* cDNA, 5′- and 3′-RACE PCRs were performed by using the SMARTer RACE cDNA Amplification Kit (Clontech) according to the manufacturer’s instructions. The primer sequences used for cloning of *TcCPR4* cDNA are shown in S1 Table. The TcCPR4 DNA sequence was deposited in GenBank (accession number KM609454).

### Real-time PCR

Total RNA isolation, first strand cDNA synthesis and real-time PCR were performed as described previously [21]. Total RNA was isolated from whole insects (n = 5 to 10 except for embryos) at various developmental stages from embryos to adults. The transcript levels of the *T*. *castaneum* ribosomal protein S6 (*TcRpS6*) were measured to normalize for differences between the concentrations of cDNA templates. See S1 Table for the primer sequences used for real-time PCR experiments.

### RNA interference (RNAi)

The template for making double-stranded RNA for *TcCPR4* (ds*TcCPR4*) was amplified by PCR using the primer set shown in S1 Table. ds*TcCPR4* (258 bp) was synthesized using an AmpliScribe T7-Flash kit (Epicentre Technologies, Madison, WI) according to the manufacturer’s protocol. ds*TcCPR4* (100 or 200 ng per insect) was injected into the dorsal abdomen of late-stage larvae (a mixture of penultimate instar and last instar larvae) (n = 20) [33]. dsRNA for the *T*. *castaneum Vermilion* gene (ds*TcVer*) was synthesized and injected to serve as a negative control [22,34]. To analyze the knockdown level of *TcCPR4* transcripts after RNAi, total RNA was isolated from whole insects (5 d-old pupae) (n = 3). Total RNA was independently isolated for each of the three replications and significant differences were analyzed using the Student *t*-test.

### Expression and purification of recombinant TcCPR4 protein

The coding sequence excluding the putative signal peptide of *TcCPR4* was amplified from the cloned cDNA by PCR using the primer set shown in S1 Table. The forward and reverse primers contain *NcoI* and *HindIII* recognition sites (underlined), respectively, to facilitate directional cloning into the pET28a expression vector (Novagen). The PCR product was digested with *NcoI* and *HindIII* and subcloned into the same sites of the pET28a plasmid DNA, and then transformed into *E*. *coli* BL21 (DE3). The recombinant TcCPR4 protein (rTcCPR4) was obtained by induction with 1 mM IPTG for 5 h at 37°C. Cells were collected by centrifugation at 3,000 x g for 15 min at 4°C, resuspended in phosphate buffered saline (0.01 M PBS, pH 7.4) containing protease inhibitor cocktail (Thermo Scientific) and lysed by sonication. The lysate was centrifuged at 3,000 x g for 15 min at 4°C. The supernatant was applied to a Ni-NTA column (Invitrogen) equilibrated with PBS followed by washes with the same buffer containing 20 mM imidazole. Bound proteins were eluted by stepwise changes in the concentration of imidazole (50, 100, 150, 200 and 250 mM) in the elution buffer. Purified rTcCPR4 was used as the antigen to generate rabbit antiserum by Cocalico Biologicals, Inc., PA, USA.

### Western blot

Western blot analysis was performed to evaluate the specificity of the anti-TcCPR4 antiserum. Elytra were dissected from 5 d-old pupae (n = 5) that had been injected previously with ds*TcCPR4* or ds*TcVer* (200 ng per insect) in the late larval instar, homogenized in 120 μl PBS containing protease inhibitor cocktail (Thermo Scientific), and centrifuged at 13,000 x g for 2 min at 4°C. The supernatant was collected as the PBS-soluble fraction. The pellet was dissolved in 120 μl of SDS sample buffer, heated at 95°C for 10 min and then centrifuged at 13,000 x g for 2 min. The supernatant was collected as the SDS sample buffer-soluble fraction. Protein samples were analyzed by 15% SDS-PAGE followed by Coomassie Blue staining or western blotting using the anti-TcCPR4 polyclonal antibody as described previously [8].

### Immunohistochemical analysis

To analyze localization of TcCPR4 protein, immunostaining was performed as described previously [35]. Cryosections (10 μm) of 5 d-old wild-type pupae or those injected with ds*TcCPR4* or ds*TcVer* at the late larval stage were prepared. Tissues were rinsed with PBST (0.01 M PBS, pH 7.4 containing 0.1% Tween 20) three times for 5 min to remove the tissue compound, and then blocked with blocking buffer (2% bovine serum albumin in PBST) for 1 h at room temperature. Then the sections were incubated with anti-TcCPR4 antibody (1:300 in 2% BSA in PBST) for 3 h at room temperature. After washing the sections with PBST three times for 5 min each, Alexa Fluor 488- or 546-conjugated goat anti-rabbit IgG (Invitrogen) secondary antibody (1:300 in 2% BSA in PBST) was added and incubated for 1 h at room temperature. After washing the sections three times with PBST, fluorescein isothiocyanate (FITC)-conjugated chitin-binding probe (1:300 in 2% BSA in PBST) was applied and incubated at 4°C for overnight. The sections were washed with PBST three times for 5 min each at room temperature and then nuclei were stained with TO-PRO-3 (Invitrogen) in PBST for 1 h at room temperature. Tissues were observed using a confocal laser scanning microscope (Olympus FV500) with appropriate filters.

### Transmission Electron Microscopy

Five day-old pupae that had been injected with ds*TcCPR4* or ds*TcVer* at the late larval stage of development were collected and fixed in a mixture of 4% paraformaldehyde and 0.1% glutaraldehyde in 0.1 M sodium cacodylate buffer (pH 7.4) for 24 h at room temperature. TEM analysis and immunogold labeling were performed as described previously [21]. For the immunogold labeling, ultrathin sectioned samples (~90 nm) were blocked with 0.01 M PBS (pH 7.4) containing 5% normal goat serum for 1 h, and then incubated with anti-TcCPR4 antibodies (1:100) in 0.05 M PBS containing 3% nonfat milk and 0.02% TWEEN 20 overnight at 4°C. The tissues were rinsed with 0.01 M PBS three times for 5 min each and 0.05 M TBS (Tris-buffered saline) (pH 7.6) three times for 5 min each at room temperature followed by incubation with the secondary antibody conjugated with 10 nm gold particles (1:20) (goat anti-rabbit IgG conjugated with 10 nm gold particles, Electron Microscopy Sciences) in 0.05 M TBS (pH 8.0) containing 0.05% fish gelatin (BB International, Cardiff, UK) for 2 h at room temperature. The tissues were washed with 0.05 M TBS five times for 5 min each, deionized water three times for 5 min each at room temperature, and then stained with 4% aqueous uranyl acetate for 10 min.

## Supporting Information

S1 Fig Developmental expression profiles of *TcCPR4*. (A) The cDNAs used for real-time PCR were prepared from total RNA extracted from whole beetles at various developmental stages (embryo to mature adults). The transcript levels of the *T*. *castaneum* ribosomal protein S6 (*TcRpS6*) were measured to normalize for differences between in the concentration of cDNA templates. TcCPR4 gene was highly expressed at the pupal stage. E, embryos; YL, young larvae; OL, old larvae; PP, pharate pupae; P, pupae; A, mature adults. (B) To analyze the expression profiles of *TcCPR4* at later stages of development, the stages analyzed were expanded between the early pharate pupal to young adult stages. The transcript levels of *TcCPR4* dramatically increased in 3 d-old pupae and declined rapidly thereafter. Expression levels for *TcCPR4* are presented relative to the levels of expression at the earliest developmental stage analyzed (E or PP0). Data are shown as mean ± SE (n = 3). PP0, day 0–1 pharate pupae; PP1, day 1–2 pharate pupae; P0, day 0 pupae; P1, day 1 pupae, P2, day 2 pupae; P3, day 3 pupae; P4, day 4 pupae; P5, day 5 pupae; A0, day 0 adults; and A7, day 7 adults.
(TIF)

S2 Fig Characterization of the TcCPR4 polyclonal antibody. Protein extracts of elytra (5 pairs) from 5 d-old pupae that had been injected with ds*TcVer* or ds*TcCPR4* (200 ng per insect) at the late larval stage were analyzed by 15% SDS-PAGE, Commassie blue staining (left panel) and western blotting (right panel). Western blotting showed that the TcCPR4 antibody detected two proteins, one with the expected mass of 22 kDa and another smaller protein (16 kDa) in the ds*TcVer* protein extract but not in the ds*TcCPR4* extract. The purified recombinant TcCPR4 protein (24.5 kDa, 40 ng) was used as a positive control.
(TIF)

S3 Fig *TcCPR4*-EGFP expression in enhancer trap line KS217. Shown are ventral, lateral and dorsal views of a pharate adult (5 d-old pupae) of an enhancer trap line, KS217 (left panels), in which a piggyBac element was inserted in a gene encoding TcCPR4, and a dissected elytron and hindwing (right panel). EGFP is expressed in cuticles of the elytron, head, pronotum, ventral abdomen and leg, all of which become highly sclerotized and pigmented in mature adults. EGFP was not expressed in flexible and less pigmented cuticles such as those of the hindwing and dorsal abdomen.
(TIF)

S4 Fig Localization of TcCPR4 and TcCPR27 proteins in thoracic cuticle. Locations of TcCPR4 and TcCPR27 proteins in thoracic body wall were analyzed by immunohistochemistry. Cryosections of 3 d- (P3), 4 d- (P4) and 5 d- (P5), old pupae were incubated with the anti-TcCPR4 or anti-TcCPR27 antibody [21]. Anti-TcCPR4 and anti-TcCPR27 antibodies were detected by Alexa Fluor 546 goat anti-rabbit IgG (red) and Alexa Fluor 488 rabbit anti-chicken IgG (green), respectively. Nuclei were stained with To-Pro-3 (blue). T: thoracic cuticle, EC: epithelial cell. Scale bar = 20 μm.
(TIF)

S5 Fig Localization of TcCPR4 and TcCPR27 proteins in the PCFs surrounding the APMPs. Ultra-thin sections (~90 nm) of wild-type pharate adults (5 d-old pupae) (A) or RNAi-treated pharate adults (5 d-old pupae) that had been injected with either ds*TcCPR4* or ds*TcVer* (100 ng per insect) in the late larval stages were prepared for immunogold labeling TEM analysis. In (A), the sections were incubated with anti-TcCPR4 or anti-TcCPR27 antibodies [21]. Anti-TcCPR4 and anti-TcCPR27 antibodies were detected using goat anti-rabbit conjugated to 10 nm gold particles and goat anti-chicken conjugated to 6 nm gold particles, respectively. Both proteins are present in the PCFs and around the APMP of the dorsal elytral cuticle. Scale bar = 200 nm. In (B), the sections were incubated with anti-TcCPR4 antibody, which was then detected by goat anti-rabbit conjugated to 10 nm gold particles. The number of gold particles is drastically decreased in TcCPR4-deficient insects (right panels), indicating that anti-TcCPR4 antibody specifically recognized the TcCPR4 protein. Scale bar = 500 nm. APMP, apical plasma membrane protrusion.
(TIF)

S6 Fig Immunolocalization of TcCPR4 protein in elytral cuticle. Cryosections prepared from pharate adults (5 d-old pupae) that had been injected with ds*TcCPR4* or ds*TcVer* in late larval stage were incubated with the anti-TcCPR4 antibody, which was then detected by Alexa Fluor 546 goat anti-rabbit IgG (red). Cuticular chitin was stained with an FITC-conjugated chitin-binding probe [35]. Nuclei were stained with To-Pro-3 (blue). E: elytron, H: hindwing, T: thoracic cuticle, D: elytral dorsal cuticle, V: elytral ventral cuticle, PC: pupal cuticle. Scale bar = 50 μm.
(TIF)

S7 Fig Elytral defects produced by RNAi for *TcCPR4*, *TcCPR27* and *TcCPR4/27*. dsRNAs for *TcCPR4*, *TcCPR27*, *TcCPR4/27* and *TcVer* (100 ng per insect) were injected into late stage larvae (n = 50). Elytral defects of the resulting adults were placed in 4 categories as follows: 1 (blue), wrinkled, fenestrated and does not cover their dorsal abdomen; 2 (red), expanded but split; 3 (green), rough surface but entire abdomen covered, 4 (purple), smooth and entire abdomen covered. Numbers in parentheses above each bar graph indicate the percentage of live adults 7 days after eclosion.
(TIF)

S8 Fig Ultrastructure of thoracic body wall and rigid leg cuticle from *TcCPR4*, *TcCPR27*- and *TcCPR4/27*-deficient insects. Ultrastructure of thoracic body wall (top panels) and leg (bottom panels) cuticle from pharate adults (5 d-old pupae) that had been injected with dsRNA for *TcCPR4* (B, F, J and N), *TcCPR27* (C, G, K and O), *TcCPR4/27* (D, H, L and P) and *TcVer* (A, E, I and M) in the late instar larvae was analyzed by TEM. The ultrastructural defects produced by injection of ds*TcCPR4*, ds*TcCPR27* and ds*TcCPR4/27* in thoracic and leg cuticle were very similar to those seen in the elytral dorsal cuticle, suggesting that TcCPR4 and TcCPR27 are critical for formation of rigid cuticle of *T*. *castaneum* adults. EN, envelope; EP, epicuticle; PRO, procuticle; PCF, pore canal fiber; APMP, apical plasma membrane protrusion. Scale bar in A-D and I-L = 1 μm and E-H and M-P = 500 nm.
(TIF)

S9 Fig Thickness of dorsal elytral cuticles from *TcCPR4*, *TcCPR27*- and *TcCPR4/27*-deficient insects. Elytra were dissected from pharate adults (5 d-old pupae) that had been injected with dsRNA for *TcCPR4*, *TcCPR27*, *TcCPR4/27* and *TcVer* in the late instar larvae. The thickness of cuticles of the dorsal side of the elytra was measured from TEM. An asterisk indicates a significant difference in thickness between control (ds*TcVer*) and test insects (p < 0.05, t-test). Data are shown as mean ± SE (n = 7–10).
(TIF)

S1 Table Primers used in this study. (DOCX)
