# Supplementary figures and images for: Tribolium castaneum RR-1 Cuticular Protein TcCPR4 Is Required for Formation of Pore Canals in Rigid Cuticle
Source: PLoS Genet. 2015 Feb 9;11(2):e1004963. doi: 10.1371/journal.pgen.1004963 (PMC4335487; doi:10.1371/journal.pgen.1004963)

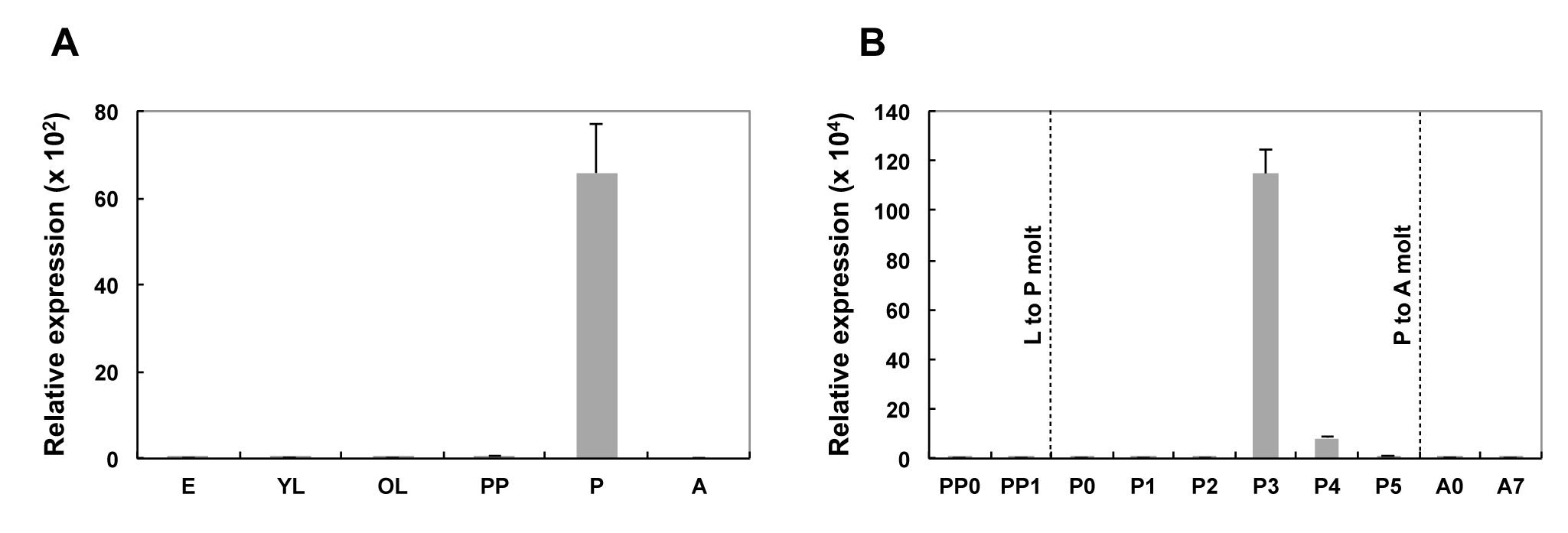

Supplement: S1 Fig — (A) The cDNAs used for real-time PCR were prepared from total RNA extracted from whole beetles at various developmental stages (embryo to mature adults). The transcript levels of the T. castaneum ribosomal protein S6 (TcRpS6) were measured to normalize for differences between in the concentration of cDNA templates. TcCPR4 gene was highly expressed at the pupal stage. E, embryos; YL, young larvae; OL, old larvae; PP, pharate pupae; P, pupae; A, mature adults. (B) To analyze the expression profiles of TcCPR4 at later stages of development, the stages analyzed were expanded between the early pharate pupal to young adult stages. The transcript levels of TcCPR4 dramatically increased in 3 d-old pupae and declined rapidly thereafter. Expression levels for TcCPR4 are presented relative to the levels of expression at the earliest developmental stage analyzed (E or PP0). Data are shown as mean ± SE (n = 3). PP0, day 0–1 pharate pupae; PP1, day 1–2 pharate pupae; P0, day 0 pupae; P1, day 1 pupae, P2, day 2 pupae; P3, day 3 pupae; P4, day 4 pupae; P5, day 5 pupae; A0, day 0 adults; and A7, day 7 adults. (TIF) [file pgen.1004963.s001.tif]

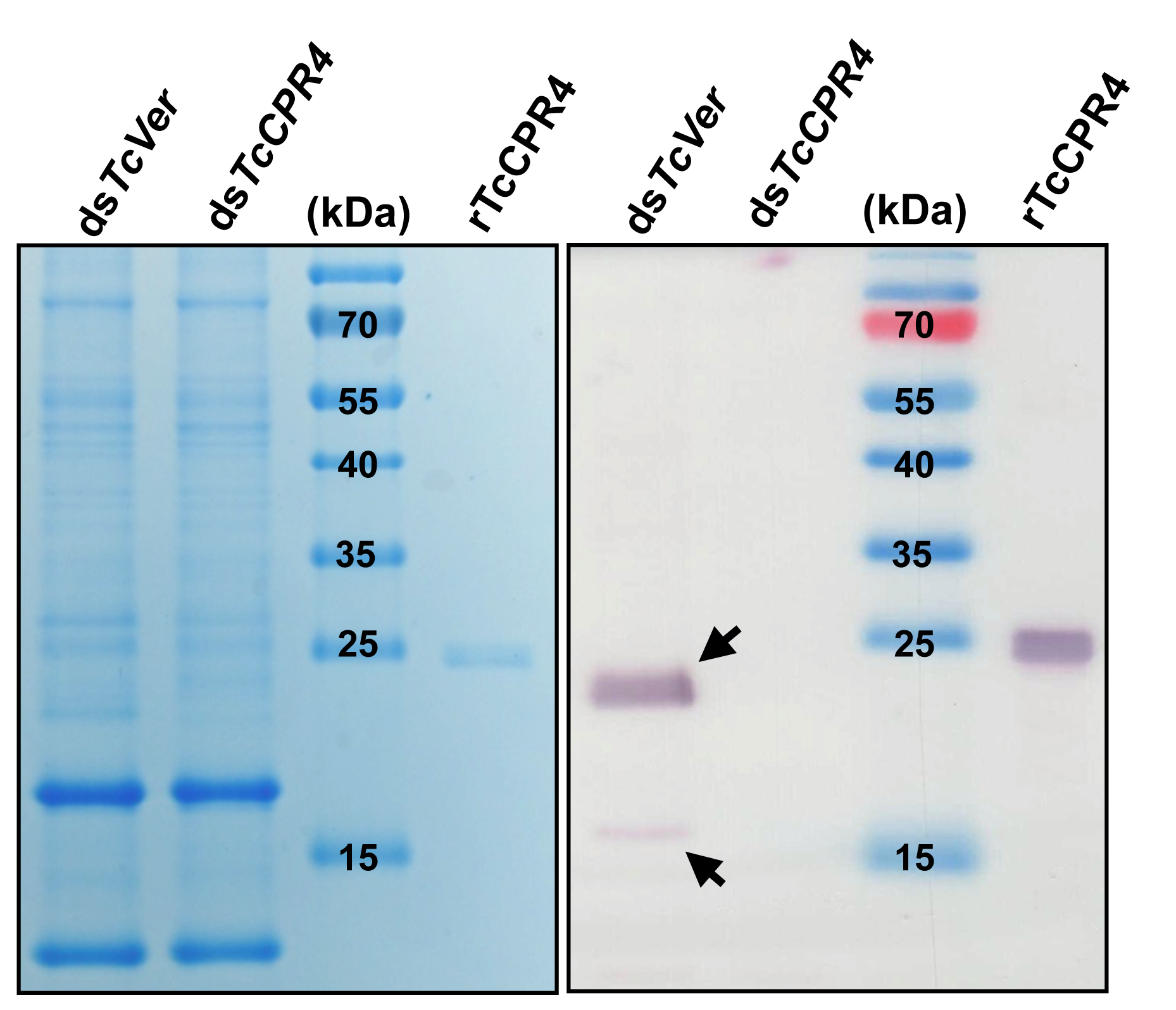

Supplement: S2 Fig — Protein extracts of elytra (5 pairs) from 5 d-old pupae that had been injected with dsTcVer or dsTcCPR4 (200 ng per insect) at the late larval stage were analyzed by 15% SDS-PAGE, Commassie blue staining (left panel) and western blotting (right panel). Western blotting showed that the TcCPR4 antibody detected two proteins, one with the expected mass of 22 kDa and another smaller protein (16 kDa) in the dsTcVer protein extract but not in the dsTcCPR4 extract. The purified recombinant TcCPR4 protein (24.5 kDa, 40 ng) was used as a positive control. (TIF) [file pgen.1004963.s002.tif]

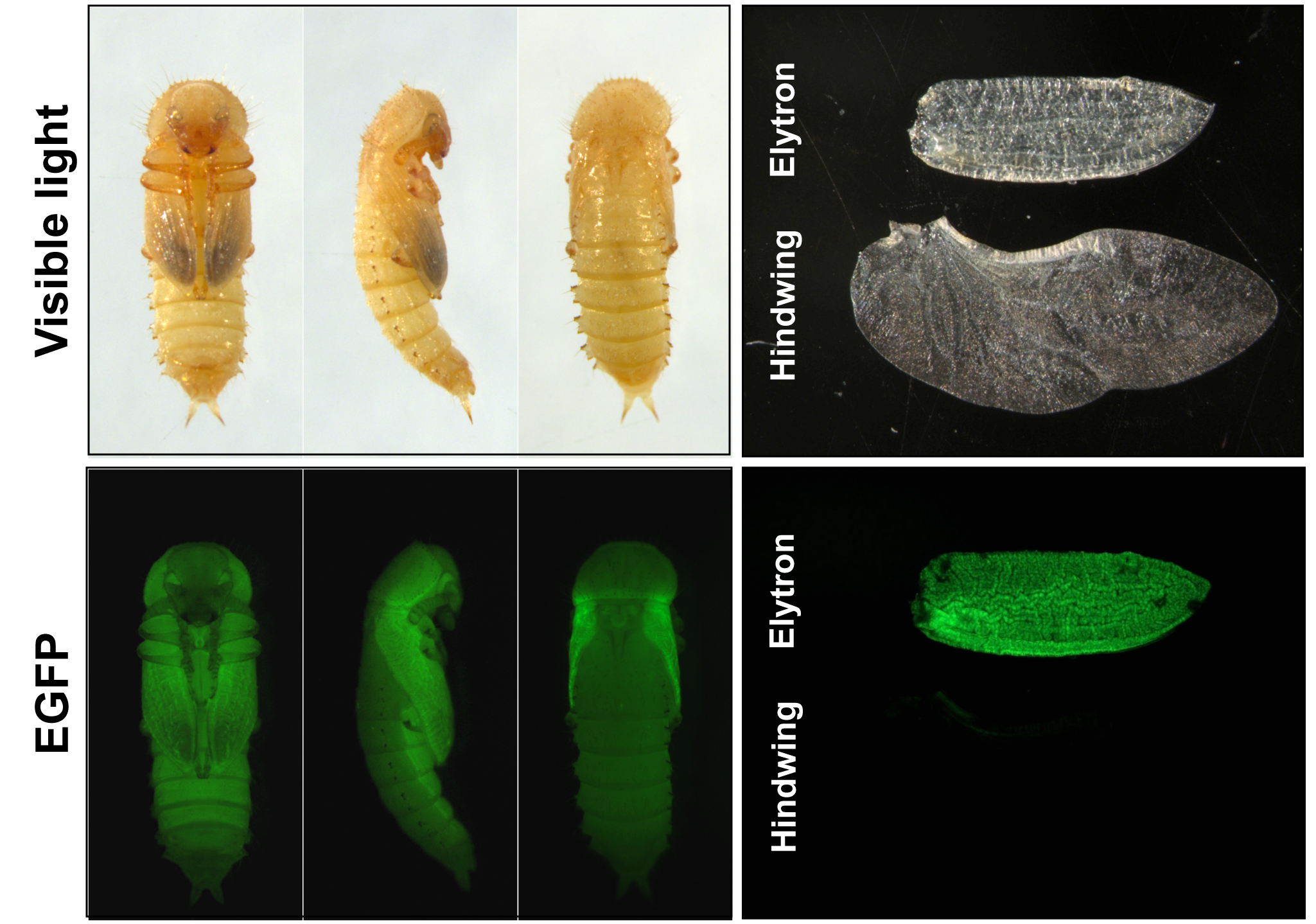

Supplement: S3 Fig — Shown are ventral, lateral and dorsal views of a pharate adult (5 d-old pupae) of an enhancer trap line, KS217 (left panels), in which a piggyBac element was inserted in a gene encoding TcCPR4, and a dissected elytron and hindwing (right panel). EGFP is expressed in cuticles of the elytron, head, pronotum, ventral abdomen and leg, all of which become highly sclerotized and pigmented in mature adults. EGFP was not expressed in flexible and less pigmented cuticles such as those of the hindwing and dorsal abdomen. (TIF) [file pgen.1004963.s003.tif]

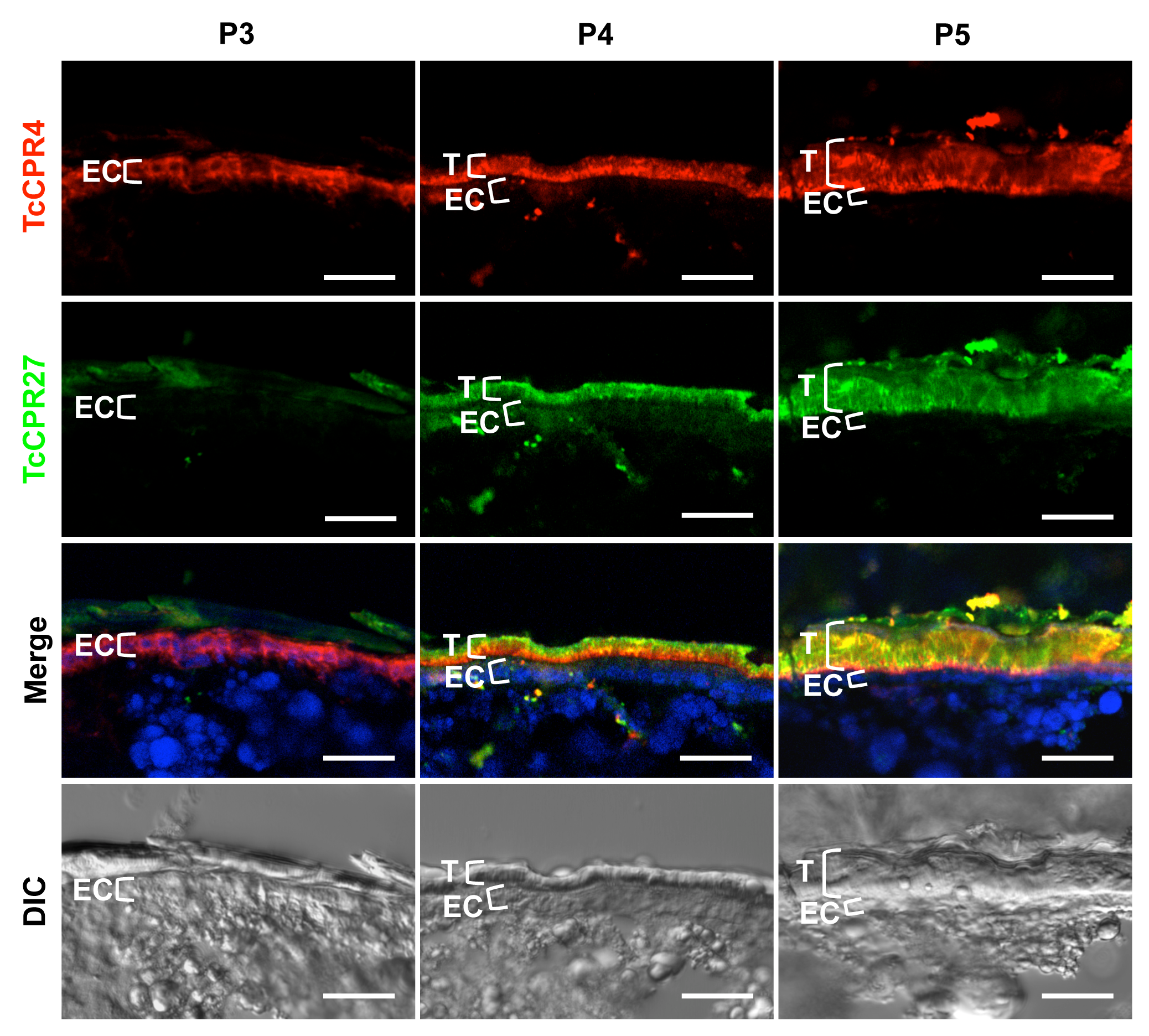

Supplement: S4 Fig — Locations of TcCPR4 and TcCPR27 proteins in thoracic body wall were analyzed by immunohistochemistry. Cryosections of 3 d- (P3), 4 d- (P4) and 5 d- (P5), old pupae were incubated with the anti-TcCPR4 or anti-TcCPR27 antibody [21]. Anti-TcCPR4 and anti-TcCPR27 antibodies were detected by Alexa Fluor 546 goat anti-rabbit IgG (red) and Alexa Fluor 488 rabbit anti-chicken IgG (green), respectively. Nuclei were stained with To-Pro-3 (blue). T: thoracic cuticle, EC: epithelial cell. Scale bar = 20 μm. (TIF) [file pgen.1004963.s004.tif]

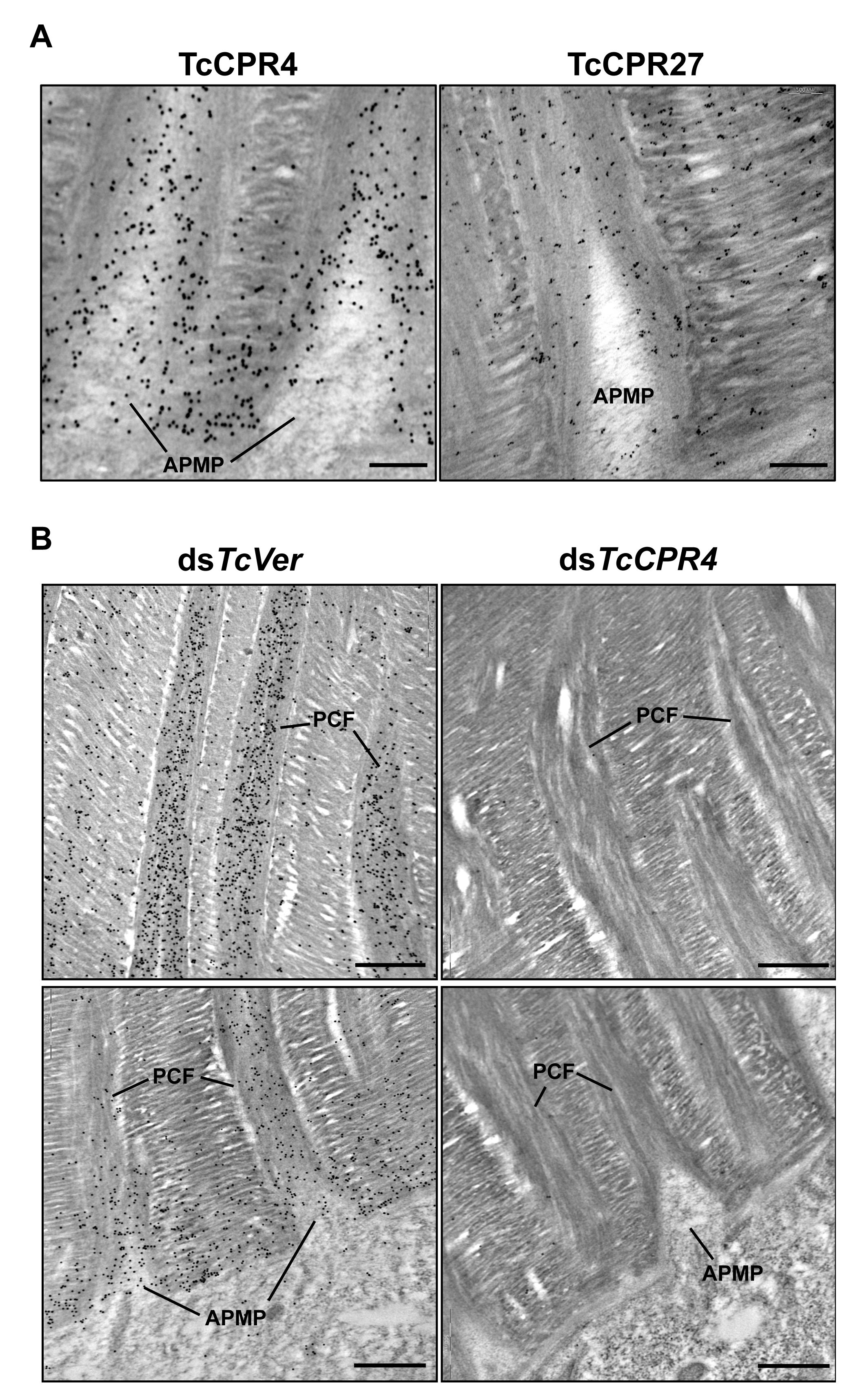

Supplement: S5 Fig — Ultra-thin sections (~90 nm) of wild-type pharate adults (5 d-old pupae) (A) or RNAi-treated pharate adults (5 d-old pupae) that had been injected with either dsTcCPR4 or dsTcVer (100 ng per insect) in the late larval stages were prepared for immunogold labeling TEM analysis. In (A), the sections were incubated with anti-TcCPR4 or anti-TcCPR27 antibodies [21]. Anti-TcCPR4 and anti-TcCPR27 antibodies were detected using goat anti-rabbit conjugated to 10 nm gold particles and goat anti-chicken conjugated to 6 nm gold particles, respectively. Both proteins are present in the PCFs and around the APMP of the dorsal elytral cuticle. Scale bar = 200 nm. In (B), the sections were incubated with anti-TcCPR4 antibody, which was then detected by goat anti-rabbit conjugated to 10 nm gold particles. The number of gold particles is drastically decreased in TcCPR4-deficient insects (right panels), indicating that anti-TcCPR4 antibody specifically recognized the TcCPR4 protein. Scale bar = 500 nm. APMP, apical plasma membrane protrusion. (TIF) [file pgen.1004963.s005.tif]

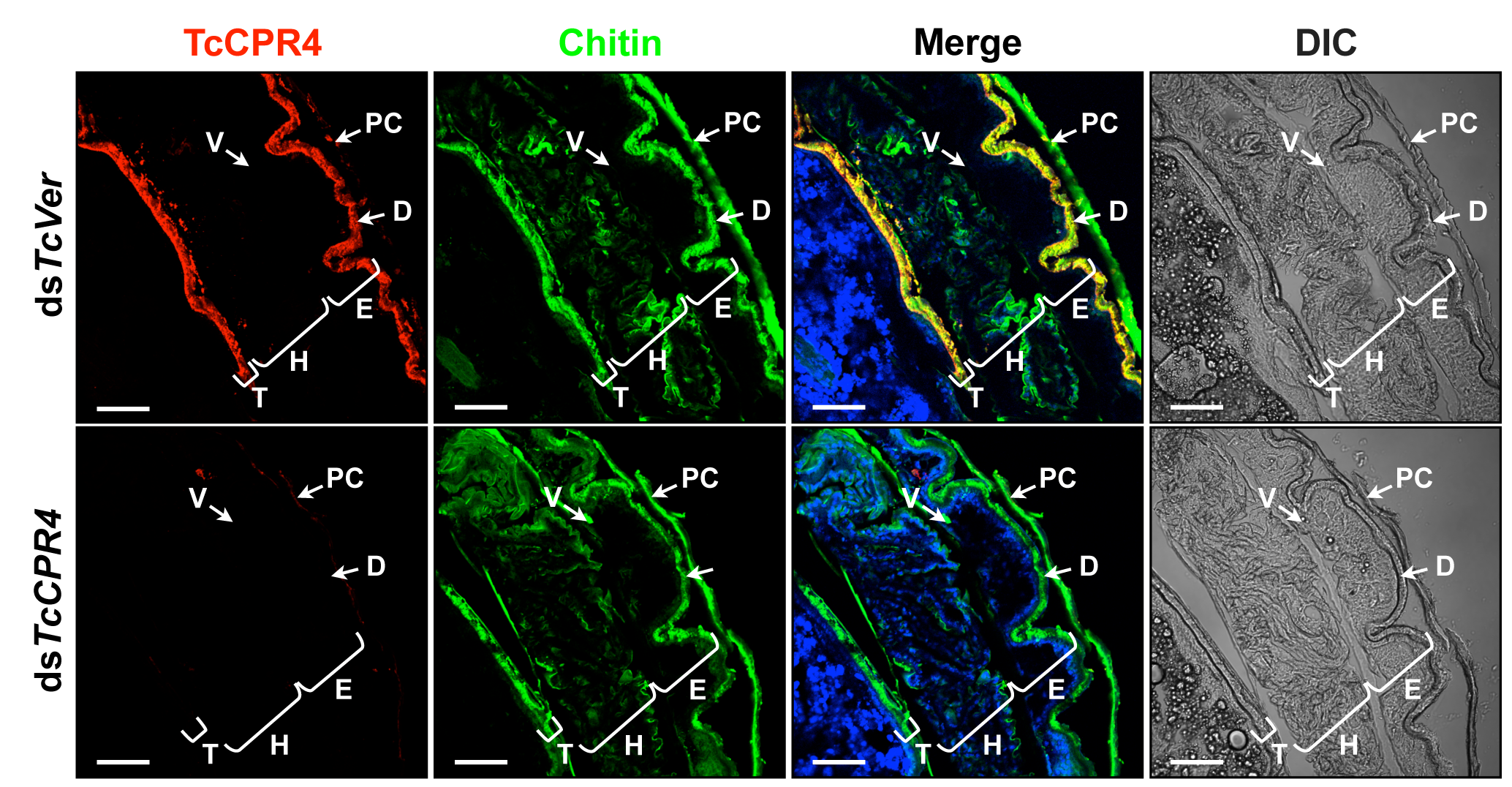

Supplement: S6 Fig — Cryosections prepared from pharate adults (5 d-old pupae) that had been injected with dsTcCPR4 or dsTcVer in late larval stage were incubated with the anti-TcCPR4 antibody, which was then detected by Alexa Fluor 546 goat anti-rabbit IgG (red). Cuticular chitin was stained with an FITC-conjugated chitin-binding probe [35]. Nuclei were stained with To-Pro-3 (blue). E: elytron, H: hindwing, T: thoracic cuticle, D: elytral dorsal cuticle, V: elytral ventral cuticle, PC: pupal cuticle. Scale bar = 50 μm. (TIF) [file pgen.1004963.s006.tif]

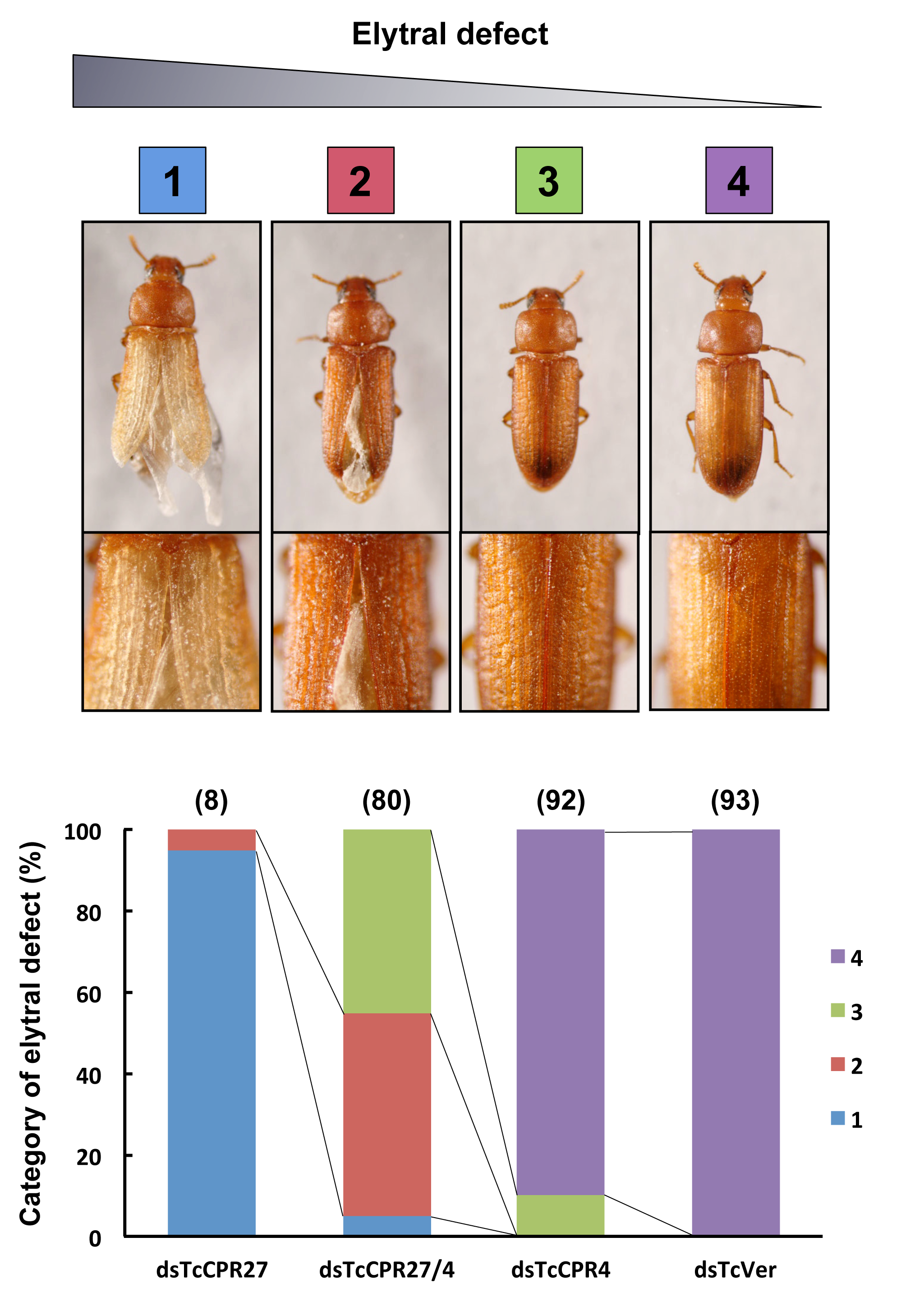

Supplement: S7 Fig — dsRNAs for TcCPR4, TcCPR27, TcCPR4/27 and TcVer (100 ng per insect) were injected into late stage larvae (n = 50). Elytral defects of the resulting adults were placed in 4 categories as follows: 1 (blue), wrinkled, fenestrated and does not cover their dorsal abdomen; 2 (red), expanded but split; 3 (green), rough surface but entire abdomen covered, 4 (purple), smooth and entire abdomen covered. Numbers in parentheses above each bar graph indicate the percentage of live adults 7 days after eclosion. (TIF) [file pgen.1004963.s007.tif]

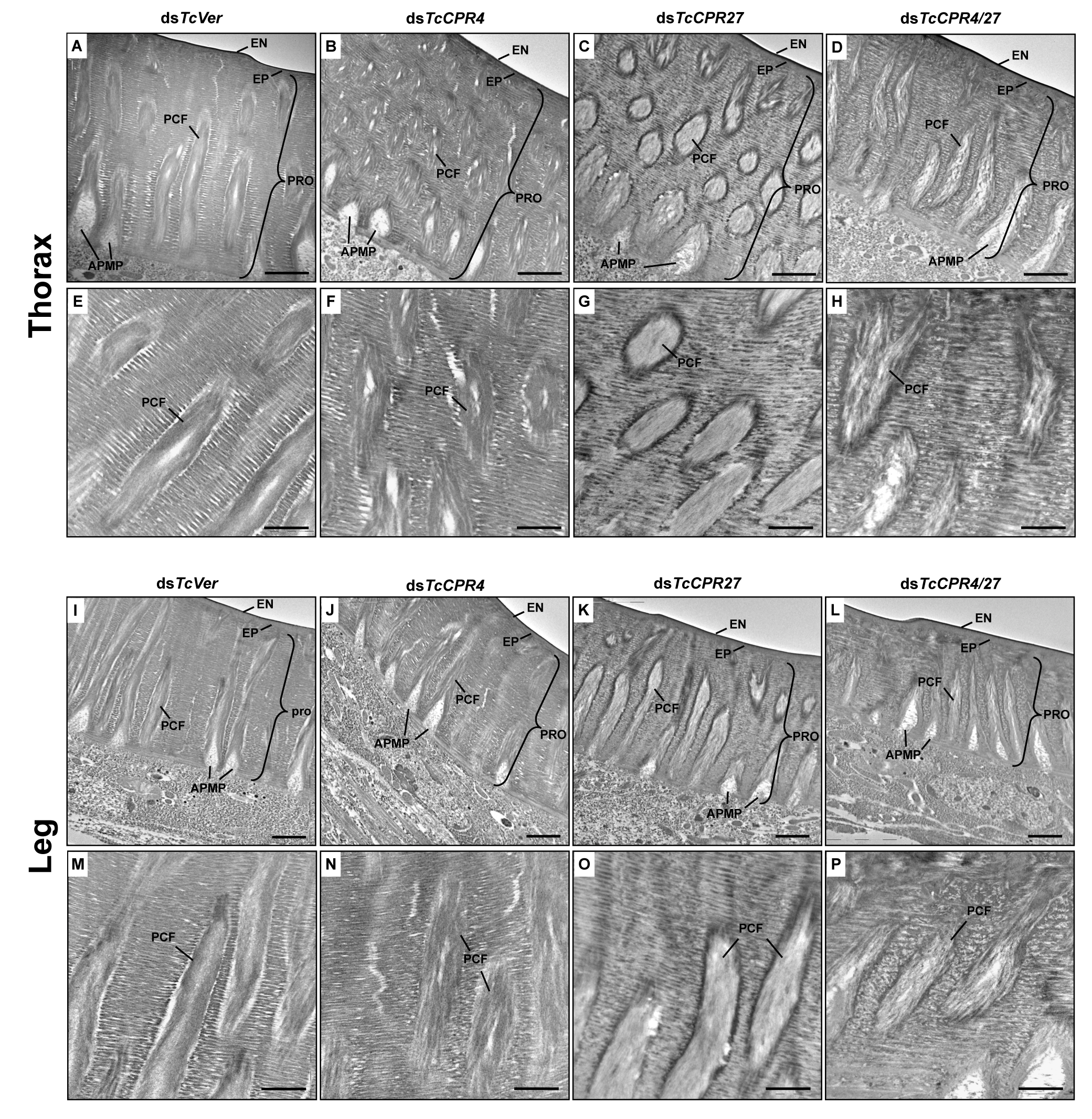

Supplement: S8 Fig — Ultrastructure of thoracic body wall (top panels) and leg (bottom panels) cuticle from pharate adults (5 d-old pupae) that had been injected with dsRNA for TcCPR4 (B, F, J and N), TcCPR27 (C, G, K and O), TcCPR4/27 (D, H, L and P) and TcVer (A, E, I and M) in the late instar larvae was analyzed by TEM. The ultrastructural defects produced by injection of dsTcCPR4, dsTcCPR27 and dsTcCPR4/27 in thoracic and leg cuticle were very similar to those seen in the elytral dorsal cuticle, suggesting that TcCPR4 and TcCPR27 are critical for formation of rigid cuticle of T. castaneum adults. EN, envelope; EP, epicuticle; PRO, procuticle; PCF, pore canal fiber; APMP, apical plasma membrane protrusion. Scale bar in A-D and I-L = 1 μm and E-H and M-P = 500 nm. (TIF) [file pgen.1004963.s008.tif]

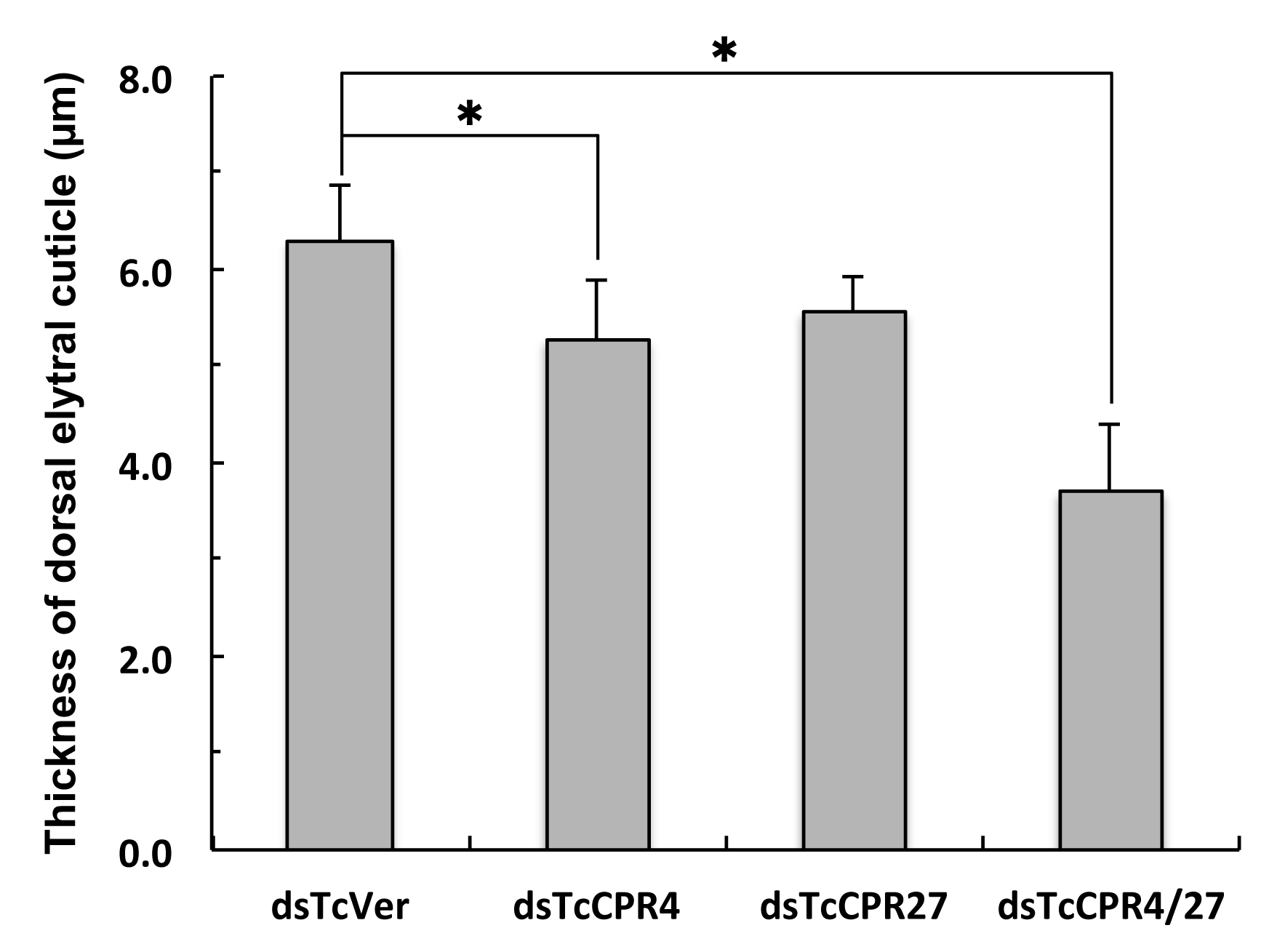

Supplement: S9 Fig — Elytra were dissected from pharate adults (5 d-old pupae) that had been injected with dsRNA for TcCPR4, TcCPR27, TcCPR4/27 and TcVer in the late instar larvae. The thickness of cuticles of the dorsal side of the elytra was measured from TEM. An asterisk indicates a significant difference in thickness between control (dsTcVer) and test insects (p < 0.05, t-test). Data are shown as mean ± SE (n = 7–10). (TIF) [file pgen.1004963.s009.tif]
