# Supplementary material for: Tribolium castaneum RR-1 Cuticular Protein TcCPR4 Is Required for Formation of Pore Canals in Rigid Cuticle
Source: PLoS Genet. 2015 Feb 9;11(2):e1004963. doi: 10.1371/journal.pgen.1004963 (PMC4335487; doi:10.1371/journal.pgen.1004963)
Supplement: S1 Table — (DOCX) [file pgen.1004963.s010.docx]

**Table S1: Primers used in this study**

| **For** | **Direction*** | | | **Primer sequence (5' - 3')** | | **Note** |
| --- | --- | --- | --- | --- | --- | --- |
| Amplification of  full-coding *TcCPR4* cDNA | | F | ATGGAATCAATCAAAATTATC | |  | |
|  |  | R | CTACCCTCCGAATTCAAACAC | |  | |
| 5’-RACE | | R | GACATAATGGAATCAATCAAAATTAT | | First PCR | |
|  |  | R | CGTTGAACCACGACGGCAATTTCA | | Second nested PCR | |
| 3’-RACE | | F | CAGTATAGGGCTGCGGTTTG | | First PCR | |
|  |  | F | TTTCCCTGCTGTGTACTTGACGGA | | Second nested PCR | |
| Real-time PCR for *TcCPR4* | | F | CGTTGAACCACGACGGCAATTTCA | |  | |
|  |  | R | TTTCCCTGCTGTGTACTTGACGGA | |  | |
| Real-time PCR for *TcRpS6* | | F | ACGCAAGTCAGTTAGAGGGTGCAT | |  | |
|  |  | R | TCCTGTTCGCCTTTACGCACGATA | |  | |
| dsRNA for *TcCPR4* | | F | (T7) GGTATTCCGGTGCAAGC | | T7: T7 polymerase recognition sequence | |
|  |  | R | (T7) CTACCCTCCGAATTCAAAC | |  |  |
| dsRNA for *TcVer* | | F | (T7) GTCTTGGTGGACCAAG | | T7: T7 polymerase recognition sequence | |
|  |  | R | (T7) CCGCCATTTCGTGATC | |  |  |
| Expression of  rTcCPR4 protein | | F | TGTCCATGGGCCAATACGGCGGCCAGGGC | | Underline: *NcoI* recognition site | |
|  |  | R | TCTCAAGCTTGCCCCCTCCGAATTCAAACAC | | Underline: *HindIII* recognition site | |

*F = forward; R = reverse
